# The impact of sex chromosome trisomies (XXX, XXY, XYY) on gaze towards faces and affect recognition: a cross-sectional eye tracking study

**DOI:** 10.1186/s11689-022-09453-x

**Published:** 2022-08-02

**Authors:** Nienke Bouw, Hanna Swaab, Nicole Tartaglia, Lisa Cordeiro, Sophie van Rijn

**Affiliations:** 1 Clinical Neurodevelopmental Sciences, Faculty of Social and Behavioral Sciences, Leiden University, PO Box 9500, 2300 RA Leiden, The Netherlands; 2 Leiden Institute for Brain and Cognition, Leiden, The Netherlands; 3 Developmental Pediatrics, University of Colorado School of Medicine, Children’s Hospital Colorado, Aurora, CO USA; 4 Department of Pediatrics, University of Colorado School of Medicine, Aurora, CO USA

**Keywords:** Sex chromosome trisomies, Young children, Eye gaze to faces, Affect recognition, Eye tracking

## Abstract

**Background:**

About 1:650–1000 children are born with an extra X or Y chromosome (47,XXX; 47,XXY; 47,XYY), which results in a sex chromosome trisomy (SCT). This international cross-sectional study was designed to investigate gaze towards faces and affect recognition during early life of children with SCT, with the aim to find indicators for support and treatment.

**Methods:**

A group of 101 children with SCT (aged 1–7 years old; M_age_= 3.7 years) was included in this study, as well as a population-based sample of 98 children without SCT (M_age_= 3.7). Eye gaze patterns to faces were measured using an eye tracking method that quantifies first fixations and fixation durations on eyes of static faces and fixation durations on eyes and faces in a dynamic paradigm (with two conditions: single face and multiple faces). Affect recognition was measured using the subtest Affect Recognition of the NEPSY-II neuropsychological test battery. Recruitment and assessment took place in the Netherlands and the USA.

**Results:**

Eye tracking results reveal that children with SCT show lower proportion fixation duration on faces already from the age of 3 years, compared to children without SCT. Also, impairments in the clinical range for affect recognition were found (32.2% of the SCT group scored in the well below average range).

**Conclusions:**

These results highlight the importance to further explore the development of social cognitive skills of children with SCT in a longitudinal design, the monitoring of affect recognition skills, and the implementation of (preventive) interventions aiming to support the development of attention to social important information and affect recognition.

## Background

About 1:650–1000 children are born with an extra X or Y chromosome, which results in the chromosomal patterns 47,XXY (Klinefelter Syndrome; KS), 47,XXX (Trisomy X or Triple X) or 47,XYY (XYY Syndrome), as compared to the typical 46,XY or 46,XX karyotype in boys and girls. These sex chromosome trisomies (SCTs) are caused by a spontaneous nondisjunction of the X or Y chromosome during early cell division and often not diagnosed [4, 6]. SCT is being increasingly identified during pregnancy as the consequence of recent technical advances of non-invasive prenatal screening (i.e., the introduction of the noninvasive prenatal screening test; NIPT). Therefore, the unique opportunity is present to gain insight in the developmental pathways and mechanisms that underlie developmental risks of very young children with SCT, an area of research that has not received much attention so far.

There is wide phenotypic variability among individuals with SCT, with an increased risk of somatic, neurodevelopmental, educational, behavioral, and psychological difficulties during development in childhood and adolescence and in adult life [37]. Neurocognitive challenges in childhood and adolescence include impairments in language development, social cognition, and executive functioning. Global intellectual functioning within SCT is variable, ranging from impaired to above average; mean intellectual functioning is in the average to low-average range [38]. However, many studies only include adolescents and adults with SCT, and a majority focus on the somatic phenotype [28]. Social adaptive functioning of individuals with SCT has recently received more attention. Although the social phenotype is variable and varies widely within the SCT group, there is increasing recognition that individuals with SCT have an increased risk for social anxiety, difficulties with social interactions and social adjustment, and impairments in social cognitive abilities [38, 41, 42]. Interestingly, neuroimaging studies in individuals with SCT have shown that the X and Y chromosomes impact brain networks involved in higher-order cognition (see for a review: [18]). A neuroimaging study comparing the impact of the extra X and Y chromosome on cortical anatomy contribute to our understanding of neural mechanisms that underlie vulnerabilities of individuals with SCT on social cognitive domain, as it was shown that the presence of an extra X or Y chromosome convergently impacts the maturation of brain areas within the “social brain” network [30].

Insights in the development of social cognition help to understand vulnerabilities in social adaptive functioning (as described in the SOCIAL model: [3]). Social cognition is defined as the ability to perceive, and process social signals, and to adequately react in social interactions [9]. These social processing skills are largely independent of other cognitive abilities, such as language, intelligence, and attention [29]. A recent review of the scarce literature on social cognitive abilities in children with SCT [39] suggests that (although these abilities are not yet fully matured) the development of social cognition, assessed by parent-report and performance-based tests, is already found to be affected from age 8 years and older. However, social cognitive functioning was not studied in younger age groups.

Social cognitive functioning results from the dynamic and complex development of brain functions and networks in the first years of life. Depending on genetic factors such as SCT, and environmental influences, brain areas involved in perceiving and understanding social information mature and facilitate social cognitive development. Difficulties with social cognition impact how children perceive and interact with their environment, which is affected in a broad range of psychopathology, including autism spectrum disorder (ASD), and attention-deficit/hyperactivity disorder (ADHD); children with SCT show higher percentages of these behavior classifications, compared to their peers (for a review see: [41]). To gain more insight into the brain-behavior dynamics leading to psychopathology, it is important to investigate age-dependent risk factors during the early development of social cognitive skills. Since social impairments have a great impact on everyday life, an objective study of social cognitive abilities during early development of children with SCT is warranted and could contribute to the identification of indicators for (preventive) support and treatment.

Social situations are rich in providing large amounts of information that need to be processed simultaneously. These situations trigger social cognitive mechanisms in individuals to select information to be able to respond adequately. Central to this selective cognitive processing of social relevant stimuli is the automatic and spontaneous visual orientation towards social information, which is referred to as social attention. Faces are especially important in the social context, as they provide a wealth of socially relevant information, and are therefore important in successful social interactions and adaptive functioning. Already from birth, newborns show an automatic orientation to faces and highly prefer to attend to face-like patterns ([21]; for a review on eye tracking studies, see [31]). Studies have shown that eye tracking is a suitable technique to assess developmental changes in different aspects of visual orientation to social important information in young children. Eye gaze to social information, as measured with eye tracking, is found to be strongly related to the ability to learn from social signals and to develop everyday social behavior [12]. Even more than other facial characteristics, the eye region is the source of information most used to understand the mental and emotional states of others, and to which we most attend [20]. In young children (and people in general), the preference to visually orient to social stimuli is largely automatic and requires little effort [24]. However, the conscious recognition of emotions on faces of others needs more processing time and other higher-order (neuro)cognitive skills are involved (such as language abilities; [1]). The recognition of affective facial expressions gives the opportunity to detect the emotional states of others and is therefore important during social interactions [15]. It is believed that impairments in social cognition (such as spontaneous visual eye gaze toward social cues and face affect recognition) may be one of the key mechanisms underlying social behavioral difficulties found in individuals with SCT (e.g. [41]).

Indeed, there is evidence that individuals with SCT attend in a different way to social cues, as compared to individuals without SCT. Eye tracking research in adult men with 47,XXY and in boys and girls with an extra X chromosome (47,XXX and 47,XXY) showed shorter fixation durations to eyes as compared to boys and girls without an extra X chromosome, and no typical tendency to first fixate on the eyes, both during the scanning of static facial expressions [40], and during dynamic presentation of faces in movie clips [45]. Studies also show that boys and adults with 47,XXY have difficulty with the recognition of facial emotions [43]. School-aged children and adolescents with an extra X chromosome (47,XXX; 47,XXY) also showed impairments in identifying angry facial expressions [45]. However, to the best of our knowledge, there have been no studies investigating whether these different processes of eye gaze to facial social information and impairments in the recognition of facial expressions also exist in very young children with SCT, and are also present in individuals with 47,XYY. For that reason, the main question of the present study is whether difficulties with eye gaze to faces and affect recognition are already present very early in life. We examined three different age groups within the 1–7-year-old age range. The reason for this was twofold. First, early childhood is a period of rapid maturation of social development at both neural, neurocognitive, and behavioral level [34]. For clinical practice, it is therefore important to investigate at what point in early childhood development proceeds differently in children with SCT as compared to peers. It is therefore crucial to study vulnerabilities in different phases of early childhood leading to risk for compromised social development in order to identify early markers for risk and targets for monitoring and intervention. Secondly, in typical development, the maturation of social skills is not linear, that is, these social cognitive abilities develop gradually and are intertwined in a temporal sequence of social milestones that may be needed to shape appropriate social functioning [34]. It is therefore important to focus on vulnerabilities in gaze towards faces and emotion recognition in different phases of early development.

Studies of reduced and deferred eye gaze towards key social emotional features in young children with other genetic syndromes, as compared to children without genetic variations (e.g. fragile X syndrome [11];) and ASD (for reviews: [7, 16]) suggest that differences with typically developing groups in processing social cues are partially determined by the nature of the task stimuli. In order to assess the nuances of eye gaze to faces in the current study, three considerations were taken into account while constructing the eye tracking paradigms. First, we studied various outcome measures: the basic ability to gaze to faces, and the choice of focal area when presented with faces for a longer period of time. Second, we studied eye gaze to faces in both static and dynamic paradigms, since it was found that individuals at risk of showing impairments with social attention perform relatively well compared to typically developing peers in tasks that use only static social stimuli, contrasted to tasks with dynamic social stimuli (see for example [13]). Last, we used paradigms with both single and multiple faces, as it has been found that social content and richness of the stimuli are significant predictors of social attention difficulties and severity of impairments in social adaptation and communication (see for example: [35]).

To summarize, school-age children, adolescents, and adults with SCT are at risk of developing difficulties in social cognitive abilities. More specifically, they show differences in directing their eye gaze to socially important cues as compared to individuals without SCT, and impairments in the recognition of facial affect expressions. Unfortunately, studies investigating the early onset and development of these parameters in very young children with SCT do not exist. A thorough investigation of eye gaze to faces and facial affect recognition skills during different age phases of early development could give more insight in the early markers and developmental pathways leading to social and communication difficulties later in life and has the potential to provide targets for (preventive) support or intervention.

In this study, we aimed to study these early markers important in social adaptive development. Our research questions were as follows: First, do children in with SCT show differences with processing social information as compared to children without SCT, i.e., attend less to socially relevant cues when looking at static faces, and dynamic social scenes in different age phases of early development? Second, do young children with SCT in different age phases have difficulties with affect recognition skills compared to their typically developing peers? Lastly, we aimed to investigate the role of research site, recruitment bias, and the role of karyotype on eye gaze to faces and affect recognition. Although many factors are involved in presentation of the SCT phenotype, such as timing of diagnosis, the aim of the current study was to contribute to the understanding of the early phenotype of SCT by focusing on eye gaze towards faces and affect recognition in different age groups, which has remained unexplored so far. Based on the relevance of the X and Y chromosomes for development of neural networks supportive of the development of social cognition, we hypothesized that young children with SCT would show different eye gaze patterns to faces and difficulties with emotion recognition, compared to their typically developing peers.

## Methods

### Participants

The present study is part of a larger ongoing longitudinal study (the TRIXY Early Childhood Study - Leiden the Netherlands), which includes children with SCT and typically developing children aged 1–8 years. The TRIXY Early Childhood Study aims to identify neurodevelopmental risk in young children with an extra X or Y chromosome. A group of 100 children with SCT (range 1–7 years old; *M*_age_= 3.69, *SD* = 1.91) was included in this study, as well as a population-based group of 98 children without SCT (42 boys; *M*_age_= 3.66, *SD* = 1.62). Mean age did not significantly differ between groups (*t* (196) = 0.11, *p* = .913). The SCT group consisted of 34 girls with 47,XXX (34%), 45 boys with 47,XXY (45%), and 21 boys with 47, XYY (21%). In order to investigate eye gaze to faces and affect recognition outcomes in different developmental stages in early childhood, the participants were divided in three age groups: children 1 and 2 years old (*n* = 61; *M*_*age*_ = 1.47 years, *SD*_*age*_ = 0.33; 32 SCT (6 47,XXX, 18 47,XXY, 8 47,XYY), 29 without SCT), children 3 and 4 years old (*n* = 83; *M*_*age*_ = 3.88, *SD*_*age*_ = 0.58; 40 SCT (13 47,XXX, 19 47,XXY, 8 47,XYY), 43 without SCT), and children 5, 6, and 7 years old (*n* = 54; *M*_*age*_ = 5.86, *SD*_*age*_ = 0.67; 28 SCT (15 47,XXX, 8 47,XXY, 5 47,XYY), 26 without SCT). To test if the frequencies of SCT types differed across age groups, a *χ* ^2^ test was conducted, and no differences were observed (*χ*^2^ (4) = 8.40, *p =* .078).

Recruitment and assessment took place at two sites: the Trisomy of the X and Y chromosomes (TRIXY) Expert Center the Netherlands, and the eXtraordinary Kids Clinic in Developmental Pediatrics at Children’s Hospital Colorado/University of Colorado in the USA. Children in the SCT group were recruited with the help of clinical genetics departments (from the Netherlands and Colorado, USA), as well as through patient-advocacy groups and social media postings. For the SCT group, recruitment bias was assessed; three subgroups were identified: (1) “Active prospective follow-up”, which included families who were actively followed after prenatal diagnosis (51% of the SCT group); (2) “Information seeking parents,” which included families who were actively looking for more information about SCT without having specific concerns about the behavior of their child (29% of the SCT group); and (3) “Clinically referred cases,” which included families seeking professional help based on specific concerns about their child’s development (20% of the SCT group).

The diagnosis of SCT was defined by trisomy in at least 80% of the cells, which was confirmed in the study by standard karyotyping. Sixty-seven children were diagnosed prenatally (65.3%; 20 girls with XXX, 32 boys with XXY, 15 boys with XYY), and 33 children postnatally (34.7%; 14 girls with XXX, 13 boys with XXY, 6 boys with XYY). Twenty-four out of 45 boys with 47,XXY received testosterone treatment (53.3%).

Children without SCT were recruited from the western part of the Netherlands and approached with information brochures about the study. All participants were Dutch (The Netherlands) or English (USA) speaking, had normal or corrected-to-normal vision, and did not have a history of traumatic brain injury. For ethical reasons, children without SCT were not subjected to genetic screening, as these children were meant to be a representation of the general population. As the prevalence of SCT is ~1 in 1000, the risk of having one or more children with SCT in group children without SCT was considered minimal and acceptable.

### Eye tracking paradigms

#### Eye gaze to static faces

**Fig. 1.**
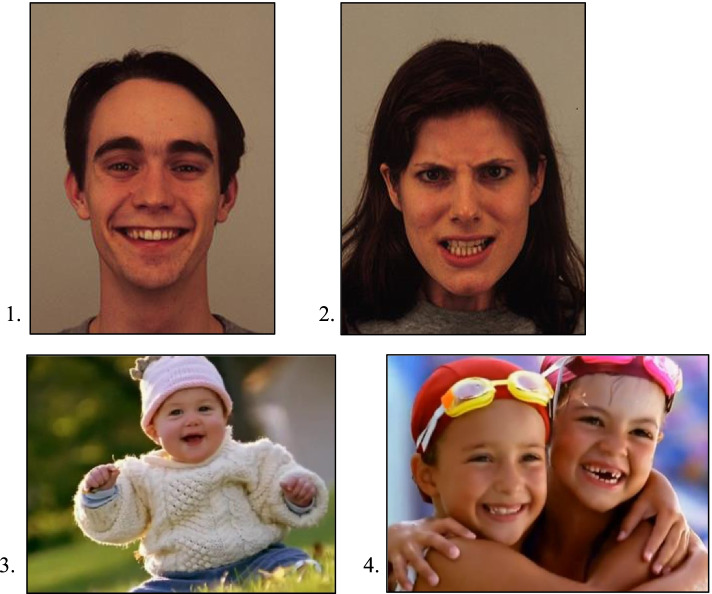
Examples of photographs in the Static Faces paradigm: (1) happy face and (2) angry face (taken from KDEF; [25]). Screenshots of video clips in the Dynamic Social Information paradigm: (3) single face and (4) multiple faces

The Static Faces paradigm consisted of 16 static photographs of cross-cultural actors with an equal distribution of two facial emotions (happy and angry), and of male and female actors (see Fig. 1). The photographs were taken from the Karolinska Directed Emotional Faces (KDEF; [25]. These KDEF pictures have no background, and actors have no visible beards, mustaches, earrings, eyeglasses, or make-up. The photographs with 7.99° × 10.87° visual angle were presented to the child, displayed at the center of the screen, in a counterbalanced order. The child was exposed to each picture for 3 s, with a 2-s inter-item interval during which an attention grabber (i.e., a picture of a toy or animal, together with a sound to grab the child’s attention) was presented in one of the four corners of the screen, to prevent for the automatic response to fixate at the center of the screen.

#### Eye gaze patterns to single and multiple faces

The Dynamic Social Information eye tracking paradigm consisted of two natural and dynamic conditions: single face (SF) and multiple faces (MF). Six trials were included (3 single face, 3 multiple faces) of 15 s each. The total time of the stimulus set was 90 s. The trials with 16.98° × 29.73° visual angle were presented in an alternate order (i.e., single, multiple, single, multiple, single, multiple). In each trial, a video clip was presented to the child. In the single face condition, one face of a child was on the screen; in the multiple faces condition, two or more faces were on the screen (child-child, child-adult, or child-adult-adult). The video clips consisted of subjects with different cultural backgrounds and were extracted from the TV broadcasted series “Baby Einstein” ([22]; see Fig. 1). The videos were accompanied by unsynchronized classical instrumental music, and no speech was involved. As these eye tracking paradigms did not involve language and used age-appropriate stimuli, it was considered to be appropriate for participants in both countries. In a group of non-clinical young children aged 3–7 years, this eye tracking paradigm was found to be significantly predictive of real-life social behaviors, and independent of age, IQ, or gender [46].

### Eye tracking equipment and procedures

Gaze data within specific areas of interest (AOIs) was collected using the Tobii X2-60 eye tracker (Tobii Technology AB, Danderyd, Sweden), which records the X and Y coordinates of the child’s eye position at 60 Hz by using corneal reflection techniques. The 15.6″ computer screen with 1080 × 1920 resolution (visual angle = 16.98° × 29.73°) with eye tracker was placed on a table adapted to the height of the seat, and the child was seated in a car seat at 65-cm viewing distance which is within the ideal range for recording, according to the Tobii X2-60 manual. A 5-point calibration procedure was used, with successful calibration defined as a maximum calibration error of 1° for individual calibration points (i.e., < 1 cm at a distance of 65 cm from the eye tracker). After the calibration procedure, the child was instructed to watch the movie clips and pictures on the computer. The two eye tracking paradigms started with an attention grabber (e.g., a moving picture of an animal, shown on a black background and accompanied by a sound) to direct the attention of the child to the screen.

Gaze data was processed using Tobii Studio (version 3.2.1), using the Tobii Identification by Velocity Threshold (I-VT) fixation filter. This filter controls for validity of the raw eye tracking data making sure only valid data were used [27]. The I-VT Threshold filter was set to define the minimum fixation duration to 60 ms, with a velocity threshold of 30°/s. Data were considered valid and were included in analysis if one or both eyes had a valid reading according to the Tobii validity criteria.

The “Dynamic AOI” tool was used to draw AOIs, drawn with a 1-cm margin, to ensure that the AOIs were sufficiently large outside the defining contours to reliably capture the gaze fixation [17]. In the Static Faces paradigm, AOIs were grouped into the category eyes (visual angle = 5.28° × 1.75°) and for the whole screen (visual angle = 16.98° × 29.73°); first fixations within the eye AOI and total fixation duration within the eye AOI were measured, in order to study eye gaze to eyes. In the Dynamic Social Information paradigm, dynamic AOIs were grouped into the following categories: face and eyes, and for the whole screen (visual angle = 16.98° × 29.73°). Total fixation duration within AOIs were measured in two conditions: single face condition and multiple face condition. In order to evaluate the amount of nonvalid eye tracking data, the total visit duration toward the whole screen was calculated, divided by the duration of the clip, multiplied by 100, reflecting the percentage of valid data collected during each of the eye tracking tests. For both paradigms, proportion fixation duration were calculated by taking the total fixation duration within the AOI, divided by the total visit duration toward the whole screen of the individual child, multiplied by 100, reflecting the percentage of time children were attending to an AOI. In the facial emotion paradigm, proportion first fixations within the AOI eyes were calculated by taking the number of photographs where participants fixated first on the eyes, divided by the total number of photographs (max = 16).

### NEPSY Affect Recognition

The Affect Recognition subtest of the Developmental NEuroPSYchological Assessment, second edition (NEPSY-II neuropsychological test battery; [23]) was designed to assess children’s ability to discriminate among common facial emotions from photographs of children, and used in this study to measure task performance of affect recognition skills. The task has been normed with typically developing children aged 3–16 years old and was administrated in a subgroup of the study sample with the age of 3 years and older (*n* = 138). During the task, participants are required to match faces of different children with different cultural backgrounds who show the same emotional expressions (happy, sad, angry, disgust, fear and neutral). The participant indicates if two expressions are the same or different, determines which two faces have similar expressions, or identifies two children with expressions that match a third child’s face. The total raw score range is between 1 and 25, with higher scores reflecting a better ability to recognize facial expressions. Besides raw scores, percentile scores as compared to norms from the general population can be calculated. Dependent upon the spoken language of the child, the Dutch or English norms were used. Percentile scores were labeled as being in the average range (percentile score > 25), the borderline range (11 < percentile score > 25), the below expected level (3 < percentile score > 10), and the well below expected level (percentile score ≤ 2).

### Cognitive assessment

To measure global level of intelligence and language three tests were administrated. The Bayley-III (subscale cognitive scale; [2]) was administered to children with the age of 1–2 years old. In the older children four subtests of the Wechsler Preschool and Primary Scales of Intelligence, 3rd edition (WPPSI-III) were used to estimate global level of intelligence (children aged 3 years: Block Design, Receptive Vocabulary, Information, Object Assembly; children aged 4 years and older: Block Design, Matrix Reasoning, Vocabulary, and Similarities; [47]). For children aged 4 years and older, total IQ estimates were calculated based on this short form version of the WPPSI-III [19]. The Peabody Picture Vocabulary Test (PPVT-III; [10]) was used to measure receptive language level in children aged 3 years and older.

### Study procedures

Assessment took place at various sites (Colorado, USA, and the Netherlands) either in a quiet room at the University (lab assessment) or at home (home assessment). To standardize the testing environment, the testing set-up and research protocols were identical at all sites. Researchers from Leiden University were responsible for project and data-management (i.e., training and supervision of researchers processing and scoring of data). Administration of cognitive and language assessment and the NEPSY was performed on a table by trained child psychologists or psychometrists in Dutch or English (dependent on the first language of the child). The eye tracking procedure took place during a separate appointment, within 1 week after the NEPSY administration. The laptop with the eye tracker was placed in a small tent to standardize the testing environment, and to control for lighting conditions. The child was seated in a car seat in front of the eye tracker. The examiner was seated beside the child (directing Tobii Studio with a remote keyboard) and started the calibration procedure. Eye tracking paradigms were shown in a fixed order (single/multiple faces, static faces). Parents were allowed to stay in the room (out of sight) and were asked not to communicate with their child during the procedure.

### Data analyses

Statistical Package for the Social Science (SPSS) version 25 was used for statistical analyses. A *χ* ^2^ test was used to compare the distribution of karyotypes within the three age groups. Pearson’s correlation analyses were used to measure the association between main outcome variables (i.e., eye gaze to faces and affect recognition) and global cognitive functioning and receptive language abilities. For group wise (SCT vs. children without SCT) comparisons of proportion first fixations, proportion duration fixation within the AOIs in the three age groups, and affect recognition skills in two age groups (M)ANOVAs were used. Pillai’s trace was used to assess the multivariate effect. Significant multivariate effects were post hoc analyzed with univariate ANOVAs to determine the locus of the multivariate effect. Influence of karyotype accounting for the effect of age was tested by an MANCOVA. (M)ANOVAs were used to investigate differences between recruitment groups, and influence of research sites was analyzed with independent *t*-tests. Statistical analyses were performed one-tailed (SCT vs. children without SCT) or two-tailed (influence of karyotype/recruitment bias/research site), and level of significance was set at *p* < .05. In case of significant differences, Cohen’s *d* or partial η2 were used to calculate effect sizes.

## Results

### Eye tracking: data quality

#### Eye gaze to static faces

The Static Faces paradigm was successfully completed by 181 children (18 children were not able to complete the task due to technical issues or fatigue of the child). Valid data were ensured by screening attention to the screen on a stimulus to stimulus basis, and stimuli of <30% attended were omitted from calculation of the average looking time for each individual child. After screening, the total proportion valid on-screen visit duration (averaged across conditions) was 83.3% and did not significantly differ between children with and without SCT, *t* (179) = −1.10, *p* = .272. Proportion first fixation and proportion fixation duration to eyes were not correlated to global cognitive functioning (respectively: *r* = .119, *p* = .114; *r* = .143, *p* = .058) and were not different between lab and home assessments (respectively: *t* (179) = −1.83, *p* = .069; *t* (179) = −.60, *p* = .549). See Table 1 for descriptive statistics for all outcome measures in the SCT and typically developing group.

**Table 1:** Descriptive statistics for SCT and TD group

	Age group	***N***	Missing	Condition	% valid data	SCT		TD		***F*** (df), ***p*** and effect size
						Min-Max	*M (SD)*	Min-Max	*M (SD)*	
Cognitive development *Norm score; Bayley-III*	1–2 years	60	1			80–125	100.39 (12.18)	72–129	99.48 (14.32)	*F*(1,58) = 0.70, *p* = .793; η_p_^2^= .01
Total IQ *WPPSI-III*	3–7 years	131	7			55–138	95.48 (19.88)	72–140	109.01 (13.27)	*F*(1,129) = −21.37, *p* < .001; η_p_^2^= .14
Receptive language *Standard score; PPVT-III*	3–7 years	135	3			65–129	99.26 (14.94)	74–133	108.67 (12.44)	*F*(1,133) = −15.86, *p* < .001; η_p_^2^= .11
Eye gaze to static faces *Proportion first fixations*	1–7 years	181	18		83.3% (angry condition: 78.5%; happy condition: 88.1%)	.00–1.00	.58 (.24)	.00–1.00	.63 (.27)	*F*(1,179) = −1.83, *p* = .089; η_p_^2^= .01
Eye gaze to static faces *Proportion fixation duration*	1–7 years	181	18			.00–.69	.32 (.17)	.00–.73	.35 (.20)	*F*(1,177) = −0.81, *p* =.185; η_p_^2^= .01
Eye gaze patterns to single and multiple faces *Proportion fixation duration*	1–7 years	188	11	SF: face	85.3%	.01–.79	.46 (.17)	.04–.83	.51 (.19)	*F*(1,186) = −4.66, *p* = .016; η_p_^2^= .02
				SF: eyes		.00–.59	.21 (.13)	.00–.61	.23 (.15)	*F*(1,186) = −1.48, *p* =.113; η_p_^2^= .01
				MF: faces	81.5%	.04–.79	.51 (.16)	.04–.80	.57 (.19)	*F*(1,186) = −5.35, *p* = .011; η_p_^2^= .03
				MF: eyes		.00–.45	.14 (.11)	.00–.57	.18 (.13)	*F*(1,186) = −4.56, *p* = .017; η_p_^2^= .02
Affect recognition *Raw score; NEPSY-II*	3–7 years	130	8			1–23	12.07 (4.58)	1–23	13.64 (4.86)	*F*(1,128) = −3.58, *p* = .031; η_p_^2^= .03

*SCT* sex chromosome trisomy, *TD* typically developing, *SF* single face, *MF* multiple faces

#### Eye gaze patterns to single and multiple faces

The Dynamic Social Information paradigm was successfully completed by 188 children (11 children were not able to complete the task due to technical issues or fatigue of the child). Total proportion valid on-screen visit duration (averaged across conditions) was 83.4% and did not significantly differ between children with and without SCT, *t* (186) = −0.10, *p* = .921. Proportion fixation duration to eyes and faces in the single face condition was not correlated to global cognitive functioning (AOI face in single face condition: *r* = .062, *p* = .403; AOI eyes in single face condition: *r* = .104, *p* = .161). Similarly, proportion fixation duration to faces in multiple faces condition was not correlated with global cognitive functioning: *r* = .111, *p* = .135). However, proportion fixation duration to eyes in the multiple faces condition was related to global intellectual functioning, *r* = .169, *p* = .022. Outcome measures were not different between lab and home assessments (SF faces: *t* (186) = 0.53, *p* = .594; SF eyes: *t* (179) = −.0.77, *p* = .445; MF faces: *t* (179) = −.036, *p* = .723; MF eyes: *t* (179) = −1.75, *p* = .081). See Table 1 for descriptive statistics for all outcome measures in the SCT and typically developing group.

### Eye gaze to static faces: age dependent group differences

#### Proportions of first fixations on eyes

Age-dependent SCT vs. typically developing group differences in first tendency to look at eyes were analyzed, when presented with static photographs of faces. Three separate ANOVAs in the three age groups were carried out with two groups (SCT vs. children without SCT) on proportions of faces where participants first fixated on the eyes. No significant effects of group (SCT vs. children without SCT) were found in the 1–2-year-old group (*F* (1,49 = 0.169, *p* = .342), and the 3–5-year-old group (*F* (1,74) = 0.479, *p* = .246). A borderline group effect (SCT vs. children without SCT) was found in the 5–7-year-old group (*F* (1,52) = 2.288, *p* = .068). See Table 2 for *M* and *SDs.*

**Table 2:** Eye gaze to static faces (proportion first fixations; proportion fixation durations) in three age groups

	Phases of development											
		1, 2 years old (*n* = 51: 24 SCT, 27 TD)			3, 4 years old (*n* = 76: 34 SCT, 42 TD)			5, 6, 7 years old (*n* = 54: 28 SCT, 26 TD)				
Eye gaze to static faces	AOI	SCT *M (SD)*	TD *M (SD)*	*p*-value	SCT *M (SD)*	TD *M (SD)*	*p*-value	SCT *M (SD)*	TD *M (SD)*	*p*-value	Post hoc effect	Effect size (η_p_^2^)
*Proportion first fixation*	Eyes	.51 (.26)	.53 (.26)	.342	.60 (.24)	.65 (.29)	.246	.62 (.23)	.71 (.22)	.068		
*Proportion fixation durations*	Eyes	.35 (.19)	.30 (.22)	.192	.35 (.17)	.38 (.20)	.289	.28 (.14)	.38 (.17)	.016	SCT < TD	.09

*SCT* sex chromosome trisomies, *TD* typically developing

#### Proportions of fixations duration on eyes

Age-dependent SCT vs. typically developing group differences in eye gaze to faces were analyzed, when presented with static faces: three separate ANOVAs with two groups (SCT vs. children without SCT) were carried out on proportions of fixation duration to eyes. In the 1–2-year-old age group, no significant effect of group (SCT vs. children without SCT) was found on the proportions of fixation duration, *F* (1,49) = 0.771, *p* = .192. Also, in the 3–5-year-olds, no significant effect of group (SCT vs. children without SCT) was found on the proportions of fixation duration, *F* (1,74) = 0.314, *p* = .289. However, in the 5–7-year-olds, a significant effect of group (SCT vs. children without SCT) was found on the proportions of fixations duration for the AOI eyes (*F* (1,51) = 4.925, *p* = .016, η_p_^2^= .09): the SCT group spent less time fixating on eyes, compared to their typically developing peers. See Table 2 for *M* and *SDs.*

### Eye gaze patterns to single and multiple faces: age dependent group differences

#### Proportions of fixation duration on eyes and faces

Within each age group, differences in eye gaze to faces with one single face (Single Face condition) and multiple faces (Multiple Faces condition) were analyzed with three separate MANOVAs, using Pillai’s trace. Descriptive statistics can be found in Table 3. In the 1–2-year-olds, there was no significant effect of group (SCT vs. children without SCT) on the proportions of fixation duration for the AOIs in both the SF and MF condition, *F* (4,52) = 0.439, *p* = .390. In the 3–5-year-old age group, a significant effect of group (SCT vs. children without SCT) was found, *F* (4,72) = 2.782, *p* = .017, η_p_^2^ = .13. Post hoc ANOVA tests on the outcome variables revealed a significant group effect with a medium effect size on the proportions of fixation duration for AOI face in the SF condition such that the SCT group spent less time fixating on the face when compared to their typically developing peers. In the 5–7-year-olds, a significant effect of group was found (SCT vs. children without SCT), *F* (4,49) = 2.165, *p* = .044, η_p_^2^= .15. Post hoc ANOVA tests on the outcome variables revealed significant group effects on the proportions of fixation duration for AOI face and AOI eyes in the MF condition with a medium effect size, revealing that the SCT group spent less time fixating on faces and eyes, when compared to children without SCT.

**Table 3:** Eye gaze patterns to single and multiple faces: proportion fixation duration on eyes and faces in two conditions (single face, multiples face): outcomes in three age groups and moderated effect of age

	Phases of development													
		1 and 2 years old (*N* = 57: 30 SCT, 27 TD)			3 and 4 years old (*N* = 77: 35 SCT, 42 TD)					5, 6, and 7 years old (*N* = 54: 28 SCT, 26 TD)				
	AOI	SCT *M (SD)*	TD *M (SD)*	*p*-value	SCT *M (SD)*	TD *M (SD)*	*p*-value	Post hoc effect	Effect size (part. η^2^)	SCT *M (SD)*	TD *M (SD)*	*p*-value	Post hoc effect	Effect size (η_p_^2^)
**Single face**	**Face**	.52 (.17)	.57 (.21)	.132	.45 (.16)	.54 (.16)	.009	SCT < TD	.07	.40 (.16)	.40 (.15)	.458		
	**Eyes**	.20 (.14)	.20 (.16)	.474	.22 (.14)	.26 (.15)	.142			.20 (.12)	.22 (.10)	.225		
**Multiple faces**	**Faces**	.56 (.15)	.60 (.21)	.172	.53 (.14)	.57 (.18)	.124			.43 (.18)	.52 (.17)	.029	SCT < TD	.07
	**Eyes**	.13 (.11)	.13 (.13)	.437	.16 (.12)	.20 (.13)	.074			.14 (.09)	.19 (.13)	.033	SCT < TD	.06

*SCT* sex chromosome trisomies, *TD* typically developing, *AOI* area of interest

### Facial affect recognition: age dependent group differences

The NEPSY Affection recognition task was administered only in the group of children aged 3 years and older (*n* = 138). Eight children were not able to finish the NEPSY Affect recognition task (total *n* = 130; 61 SCT (26 children with 47, XXX; 26 children with 47, XXY; 9 children with 47,XYY), 69 without SCT). Affect recognition scores were not correlated to global cognitive functioning (*r* = .162, *p* = .071), but were correlated to receptive language skills (*r* = .604, *p* <.001). See Table 1 for descriptive statistics of all outcome variables for both the SCT and typically developing group.

**Fig. 2:**
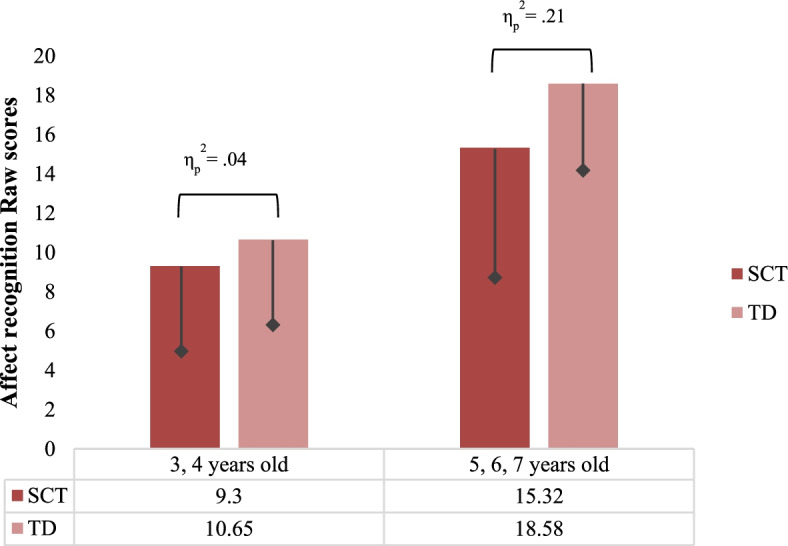
Affect recognition in SCT vs. TD group and age groups. SCT, sex chromosome trisomies; TD, typically developing. η_p_^2^, effect size;  , standard deviation (only lower bar depicted)

Within the two age groups (3–5; 5–7), differences in affect recognition were analyzed with two separate ANOVAs. Differences between the SCT group and their typically developing peers were found in both age group; see Fig. 2. When evaluating scores normalized for age, for affect recognition in the SCT group, 54.2% scored in the average level, 5.1% in the borderline range, 8.5% scored in the below expected level, and 32.2% in the well below expected level.

### Karyotype differences within the SCT group

In order to investigate the influence of various karyotypes on eye gaze to faces and affect recognition taking into account the effect of age, MANCOVAs were carried out with main effect of karyotype (XXX vs. XXY vs. XYY), and age as covariate. No differences between karyotypes were found for all eye tracking outcome measures. A significant difference between karyotypes was found for affect recognition (XXY < XXX), when age was accounted for and kept constant. See Table 4 for estimated marginal means, and *p*-values, post hoc effects and effect sizes.

**Table 4:** Differences between karyotypes on eye gaze to faces and affect recognition accounted for age (EMM (SE))

			47,XXX	47,XXY	47,XYY	***p***-value	Post hoc effect	Effect size (η_**p**_^**2**^)
**Eye gaze to faces**	**Condition**	**AOI**	*n* = 30	*n* = 41	*n* = 15			
Eye gaze to static faces *Proportion first fixations*		Eyes	.60 (.05)	.69 (.05)	.49 (.08)	.074		
Eye gaze to static faces *Proportion fixation duration*		Eyes	.29 (.03)	.40 (.03)	.30 (.05)	.056		
Eye gaze patterns to single and multiple faces *Proportion fixation duration*	Single faces	Faces	.46(.03)	.45 (.03)	.40 (.05)	.547		
		Eyes	.22 (.03)	.25 (.03)	.17 (.04)	.284		
	Multiple faces	Faces	.51 (.03)	.51 (.03)	.40 (.06)	.223		
		Eyes	.17 (.02)	.18 (.02)	.09 (.04)	.107		
**NEPSY Affect Recognition**			*n =* 28	*n =* 19	*n =* 14			
Raw score			13.56 (.67)	10.55 (.69)	11.98 (1.12)	.011	XXY<XXX	.14

*EMM* estimated marginal means, *AOI* area of interest

### Recruitment bias within the SCT group

Within the SCT group we tested with MANOVA for differences on eye gaze to faces and ANOVA for difference on affect recognition between the three recruitment groups (A: prospective follow-up, B: information seeking parents, and C: clinically referred cases group). Differences between recruitment groups were only analyzed in the study measures in which a difference was found between children with and without SCT. There were no significant differences for study outcomes between the recruitment groups (except for proportion fixation duration on faces in the dynamic social information paradigm; single faces). See Table 5 for means, exact *p*-values, post hoc effects, and effect sizes.

**Table 5:** Differences in eye gazes to faces and affect recognition across SCT recruitment groups (M, SD)

			Prospective follow-up (A)	Information seeking parents (B)	Clinically referred cases (C)	***p***-value	Post-hoc effect	Effect size (η_**p**_^**2**^)
**Eye gaze to faces**	**Condition**	**AOI**	*n* = 41	*n* = 25	*n* = 18			
Eye gaze to static faces *Proportion fixation duration*		Eyes	.32 (.17)	.35 (.19)	.33 (.15)	.851		
Eye gaze patterns to single and multiple faces *Proportion fixation duration*	Single faces	Faces	.42 (.17)	.52 (.15)	.44 (.14)	.032	A < B	0.12
	Multiple faces	Faces	.47 (.18)	.57 (.14)	.51 (.14)	.080		
		Eyes	.12 (.11)	.18 (.12)	.16 (.10)	.117		
**Affect recognition**			*n =* 28	*n =* 19	*n =* 14			
Raw scores			11.14 (4.55)	12.21 (4.69)	13.71 (4.30)	.229		

*SCT* sex chromosome trisomy, *AOI* area of interest

### The role of research site

To control for the potential impact of research site on outcomes of the study, the data of the two research sites were compared. Comparing the outcome measures in the SCT group between both research sites (the Netherlands vs. the USA), revealed a consistent pattern of results, indicating that none of the eye movement measures showed significant differences between research sites (see Table 6). However, a significant difference between research sites was found for affect recognition skills: children in the USA had lower affect recognition scores (*M=*13.11*, SD=*4.81), compared to children in the Netherlands (*M=*10.65*, SD=*3.90; *p =*.037*,* Cohen’s *d =* 0.56).

**Table 6:** Impact of research site on eye gaze to faces and affect recognition in the SCT group

	Condition	The Netherlands ***M (SD)***	USA ***M (SD)***	***t***-value	***p***-value
Eye gaze to static faces *Proportion first fixations*		.61 (.22)	.55 (.26)	1.05	.296
Eye gaze to static faces *Proportion fixation duration*		.32 (.16)	.32 (.19)	0.14	.887
Eye gaze patterns to single and multiple faces *Proportion fixation duration*	SF: face	.43 (.16)	.48 (.17)	1.46	.147
	SF: eyes	.21 (.13)	.20 (.13)	0.22	.823
	MF: faces	.50 (.18)	.52 (.15)	0.61	.545
	MF: eyes	.15 (.11)	.13 (.10)	0.92	.361
Affect recognition *Raw scores*		13.11 (.4.81)	10.65 (3.90)	2.14	.037

## Discussion

This study aimed to investigate age-dependent eye gaze to faces and affect recognition vulnerabilities in very young children with sex chromosome trisomies (SCT) aged 1–7 years. Key outcomes of the study include differences in automatically orienting and holding eye gaze to socially important information between children with and without SCT, suggesting that young children with SCT are less inclined to automatically orient towards social information. These difficulties with directing eye gaze to social important information were most pronounced in children aged 3 years and older, and when the richness of the social stimuli was high (i.e., multiple faces). Also affect recognition impairments were found, with on average 32.2% of the group children with SCT scored in the well below expected range.

First of all, we explored with the help of eye tracking measures gaze to static faces, and gaze patterns to eyes and faces in dynamic social scenes with a single face and multiple faces. Analyses in specific age groups revealed that 1–2-year-old children with SCT showed no different tendency to initially fixate on the eyes, when presented with a face. Also, when presented with dynamic social interactions, the results revealed no differences in eye gaze patterns between 1- and 2-year-olds with and without SCT (i.e., displayed no shorter fixation duration to eyes nor to face). However, in the 3–5-year-old group, we did find differences between the children with SCT and typically developing children: children with SCT were less inclined to fixate eye gaze towards faces when looking at dynamic social stimuli with a single face, although percentages of first fixation to the eye region of static faces were similar to 3–5year-olds without SCT. These results suggest that children with SCT aged 3–5 years assess static faces with emotions as fast as their typically developing peers, but are less consistent in their choice of focal area when presented with dynamic social stimuli with one single face.

Moreover, 5–7-year-olds with SCT showed lower fixation duration on eyes compared to typically developing children, when presented with static faces. In addition, children with SCT aged 5–7 years old fixated less on socially important information (both faces and eyes) when presented with dynamic social stimuli with multiple faces. This pattern of findings among 5–7-year-olds shows that differences in eye gaze to social stimuli between the SCT and typically developing group occur as a function of the richness of social information: if the richness of socially relevant and dynamic information is high (i.e., multiple faces), children with SCT deviate eye gaze from central and important social information (i.e., eyes and faces).

Taken together, these eye tracking results reveal that children with SCT generally are less inclined to automatically orient their gaze at relevant social-emotional information (i.e., eyes and faces), compared to typically developing children from preschool age on. Research has shown that typically developing children preferentially attend to social stimuli, beginning as early as infancy. Furthermore, high social content typically increases attention of children towards the eyes and faces [5]. However, our results suggest that, on average, very young children with SCT, on average, have difficulties with attention to social cues, with more impairments when presented with high richness of social information (i.e., multiple faces) as compared to on single faces, and more impairments in children of older age, as compared with their typically developing peers.

Reduced eye gaze to socially meaningful and complex stimuli already during early development, and more pronounced difficulties with eye gaze to social relevant stimuli if the amount of social information is rich, may have substantial impact on the fundamentals of social learning. Reduced eye gaze to social important information may lead to limited quantitative and qualitative opportunities to acquire social knowledge in children with SCT, and to learn from (complex) social interactions [26]. Attending to another person’s face and eyes allows typically developing children to have rich social experiences that are crucial for the development of social and communicative abilities, such as joint attention, language acquisition, and face or affect recognition [14]. Consequently, avoidance of the eyes and faces of others may have a broad impact on the complex maturation of social (cognitive) abilities, which are built upon basic social-perceptual information. Earlier studies reported social attention deficits in adult men with an extra X chromosome [40, 45]. These adult studies might represent the cumulative effects of long-term atypical visual orientation to socially important information, whereas the results of the current study suggest a developmental pathway in which profiles of impairments are emerging during early childhood.

Although such longitudinal relations between eye gaze to social important information and more complex social processing abilities not being assessed in this study, we did investigate age-dependent affect recognition skills in very young children with SCT, between the ages of 3 and 7 years. Difference was found for affect recognition abilities between children with and without SCT from the age of 3 years old, indicating deficits in young children with SCT. Earlier studies also found impairments with affect recognition in school-aged children and adolescents with SCT, in both parent report of individuals with SCT [8, 32, 44] and direct assessment of individuals with an extra X chromosome [43, 44]. Percentages of young children with SCT that scored in the clinical range in the current study (32.8%) are comparable with earlier research in older individuals with an extra X chromosome, and add to the literature that clinically significant deficits already arise early in development, and can also be found in boys with 47,XYY.

A difference between research sites was found for affect recognition abilities in children with SCT, which may suggest that cultural and social factors can be related to emotional processing. Although we acknowledge that cultural differences may contribute to some of the variance in the outcome, we are confident that this is not relevant for the systematic group differences between the SCT and typically developing group that we found in the current study. Further research could study the influence of ethnicity, cultural differences, and family environment on affect recognition abilities in children with SCT. As no differences between research sites were found for study outcomes measured with eye tracking methods, we suggest to use eye tracking methods in international studies aimed to measure the influence of culture on emotion processing.

When exploring the influence of specific karyotype (XXX, XXY, XYY) on eye gaze patterns to faces and affect recognition, accounting for the effect of age, results showed that for the majority of social cognitive measures no significant differences between the karyotypes were found. However, for affect recognition, boys with 47,XXY showed to be more vulnerable as compared to some of the other SCT karyotypes (see Table 5). These results suggest that although eye gaze patterns to faces and affect recognition were impaired in all karyotypes and older children with SCT had more difficulties than younger children, boys with XXY may be more vulnerable in their ability to recognize facial affects than other SCT karyotypes.

The results of the present study have clinical implications. Effects of chromosomal trisomies often become more apparent later on in development, when a child is faced with developmental tasks and when compromised development of the brain leads to an increasing discrepancy with the age-required norms [33, 36]. It is therefore important that social attention and affect recognition skills are included in standard neuropsychological assessment from the age of 3 years old, in addition to assessments of language and learning difficulties, to allow for close monitoring in children with SCT. Sensitive developmental periods also serve as key windows of opportunity, and early implementation of (preventive) support and intervention programs on social attention and affect recognition skills have the potential to reduce risk for social and communication impairments, and to optimize quality of life.

Regarding possible bias of recruitment on the outcomes variables, eye gaze patterns to faces and affect recognition (except for one eye tracking parameter) were not dependent on recruitment strategy, i.e., prospective follow-up group, information seeking parents group, or clinically referred cases group. These findings suggest that the outcomes of this study are representative for this group of diagnosed children with SCT as a whole. However, it remains unsure to what degree the findings in this study can be generalized to those who have SCT, but remain undiagnosed (see for example: Berglund et al. [4] for estimated proportions of underdiagnosing in SCT). This may concern children who do not require clinical care or children who do require care, but for whom it is not known that SCT is an underlying genotype.

Limitations of the current study include the cross-sectional design that limits cause-effect conclusions. Future studies should focus on the longitudinal development of social attention in children with SCT, and the impact of altered attention to social information on affect recognition and other social (cognitive) functions (e.g. Theory of Mind). In this study, we only focused on gaze towards faces with affective expressions of basic emotions, as these convey a high load of social information, more so than neutral faces. Based on our findings, it would be interesting to learn more about the impact of SCT on the scanning of faces in general. Future research should also address the questions whether intervention programs targeting the early development of affect recognition skills are effective in improving these skills and if so, whether interventions lead to improved social behavioral outcomes. As it was beyond the scope of this study to investigate the influence of testosterone treatment in boys with 47,XXY, future studies with suitable designs (e.g., Randomized Control Trials) should study these parameters in relation to general social cognitive functioning in children with SCT.

## Conclusion

In conclusion, the overall results of this study indicate that young children with SCT (on average) have difficulties automatically orienting and holding their attention to socially important information, especially when the richness of social stimuli is high (i.e., multiple faces). These difficulties with eye gaze to social stimuli were found in children with SCT aged 3 and older. In addition, impairments in facial affect recognition skills were found, with 32.8% of the SCT children scoring in the clinical range. This calls for a focus on the monitoring of social cognitive functioning from an early age onwards in SCT. These findings also highlight the importance of further exploring the developmental pathway of social attention in children with SCT in studies with a longitudinal design that allows for more understanding of the predictive value of these social cognitive skills for social behavioral difficulties and psychopathology, and the implementation of (preventive) early interventions aiming to support social cognition, to positively influence developmental outcomes in children with SCT.

## Data Availability

The datasets generated and/or analyzed during the present study are not publicly available due to confidentiality constraints within our ethical approvals.

## Abbreviations

SCT: Sex chromosome trisomies
AOI: Area of interest
